# The effect of underwater sounds on shark behaviour

**DOI:** 10.1038/s41598-019-43078-w

**Published:** 2019-05-06

**Authors:** Lucille Chapuis, Shaun P. Collin, Kara E. Yopak, Robert D. McCauley, Ryan M. Kempster, Laura A. Ryan, Carl Schmidt, Caroline C. Kerr, Enrico Gennari, Channing A. Egeberg, Nathan S. Hart

**Affiliations:** 1 Oceans Graduate School and the UWA Oceans Institute, The University of Western Australia, Perth, WA 6009 Australia; 2 Biosciences, College of Life and Environmental Sciences, University of Exeter, Exeter, EX4 4QD UK; 3 School of Life Sciences, La Trobe University, Bundoora, VIC 3086 Australia; 4 Department of Biology and Marine Biology and the Centre for Marine Science, University of North Carolina Wilmington, Wilmington, NC 28403 USA; 5 Centre for Marine Science and Technology, Curtin University, Perth, WA 6102 Australia; 6 Department of Biological Sciences, Macquarie University, North Ryde, NSW 2109 Australia; 7 Oceans Research Institute, Mossel Bay, 6500 South Africa; 8 South African Institute for Aquatic Biodiversity, Grahamstown, 6140 South Africa; 9 Department of Ichthyology and Fisheries Science, Rhodes University, Grahamstown, 6140 South Africa

**Keywords:** Behavioural ecology, Auditory system, Animal behaviour

## Abstract

The effect of sound on the behaviour of sharks has not been investigated since the 1970s. Sound is, however, an important sensory stimulus underwater, as it can spread in all directions quickly and propagate further than any other sensory cue. We used a baited underwater camera rig to record the behavioural responses of eight species of sharks (seven reef and coastal shark species and the white shark, *Carcharodon carcharias*) to the playback of two distinct sound stimuli in the wild: an orca call sequence and an artificially generated sound. When sounds were playing, reef and coastal sharks were less numerous in the area, were responsible for fewer interactions with the baited test rigs, and displayed less ‘inquisitive’ behaviour, compared to during silent control trials. White sharks spent less time around the baited camera rig when the artificial sound was presented, but showed no significant difference in behaviour in response to orca calls. The use of the presented acoustic stimuli alone is not an effective deterrent for *C. carcharias*. The behavioural response of reef sharks to sound raises concern about the effects of anthropogenic noise on these taxa.

## Introduction

Acoustic methods for modifying the behaviour of marine animals are particularly appealing since sound stimuli can propagate much further than any chemical, electrical or visual cue. However, sound-induced behavioural changes in sharks are poorly understood. Previous studies have focused on the effects of sounds on marine mammals and bony fishes, both in a conservation context^1–4^ and as a way to control an animal’s behaviour^5,6^. Anthropogenic noise in the ocean has been shown to affect foraging, vocalisation, and movement of marine mammals (e.g.^7,8^). Similarly, bony fishes have been shown to display changes in movement patterns, feeding behaviour, social interactions, and antipredator behaviour as a consequence of anthropogenic noise (e.g.^9–11^). Following these observations, sound has been used successfully as a non-physical barrier to fish movement, for example to deter them from entering the water intakes of power plants and dams (e.g.^12,13^). Similarly, Acoustic Deterrent Devices (ADD) that produce intense aversive sounds are increasingly being used as way to deter depredating species, typically pinnipeds, from aquaculture facilities^6^. Only a handful of studies in the 1960–70 s investigated the potential for attracting or deterring sharks with sound. Low frequency and pulsed sounds appear to be attractive to sharks^14–20^, whereas withdrawal behaviour has been observed in sharks exposed to orca calls and abrupt, loud, irregular sounds. Myrberg *et al*.^20^ described the withdrawal response of silky sharks, *Carcharhinus falciformis*, using playback of orca calls and an artificial irregularly pulsed noise-band (0.5–4 kHz). These results were also observed with captive lemon sharks, *Negaprion brevirostris*^21^; the amplitude of the signal and the rate at which that amplitude was achieved (onset) determined whether a shark would initiate an approach, continue its approach or suddenly withdraw from a sound source^20,21^.

The auditory apparatus of sharks comprises the paired inner ears that, as in all fishes, detect the particle motion component of a sound. Unlike most bony fishes however, cartilaginous fishes do not possess a swim bladder, which responds to the pressure component of a sound, and therefore are thought to only be sensitive to particle motion. Two sensory maculae, the sacculus and the macula neglecta, have been shown to be responsive to particle motion detection in the inner ear of cartilaginous fishes^22,23^, which are known to be sensitive to low frequency sounds up to 1.5 kHz, peaking between 200 and 600 Hz, depending on the species^24–36^. Ryan *et al*.^37^ presented artificially constructed sounds of varying frequencies (from 20 Hz to 20 kHz) and intensities (SPL 114–157 dB re 1 µPa) to captive Port Jackson (*Heterodontus portusjacksoni*) and epaulette (*Hemiscyllium ocellatum*) sharks, and to wild white sharks (*Carcharodon carcharias*). The artificial sound playback did not directly elicit any changes in the feeding behaviour of these species, possibly due to the choice of sound and its frequency and amplitude range, in addition to limitations imposed by a laboratory environment for acoustic behavioural studies, at least in the two captive species. However, there are published accounts^38,39^ of sharks being attracted by sounds from hundreds of metres away (i.e. by prey, boats, and swimmers), which contradicts our current understanding of the shark’s auditory system beyond the near-field proprieties of particle motion. This highlights the paucity of knowledge and the associated misconceptions of the effects of sounds on shark behaviour.

Orca (*Orcinus orca*) are known to prey on cartilaginous fishes, including large sharks and rays^40–44^. They are highly vocal and mostly produce pulsed calls, in addition to whistles and echolocation clicks^45,46^. The pulsed calls exhibit a complex frequency and time structure, between 500 Hz and 25 kHz and lasting 0.5–1.5 s, and can dissipate over long distances with source levels of 131–176 dB re 1 µPa at 1 m root-mean-square^45,47^. Most importantly, while some calls may possess a high-frequency component (HFC), the pulsed calls always contain a low-frequency component (LFC)^48,49^. The LFC is composed of multiple temporal sequences separated by shifts in the pulse repetition rate, and contain frequencies lower than 3 kHz^48,50,51^, thus overlapping the currently described shark hearing frequency range. Specific avoidance to orca sounds have been demonstrated in teleosts^52^, pinnipeds^53^ and some other cetacean species^54–57^, as well as bony fishes^58^. Myrberg *et al*.^20^ demonstrated withdrawal of silky (*C. falciformis*) and oceanic whitetip sharks (*Carcharhinus longimanus*) from sounds with a loud onset of noise between 150 and 600 Hz. The abrupt change in amplitude level, rather than the actual nature of the sound (i.e. orca call), appeared to trigger the flight response, although the authors could not ascertain whether changes in temporal or spectral attributes of the sound were alone sufficient to elicit withdrawal^20^. These results were confirmed in a study with captive lemon sharks, *N. brevirostris*, which were deterred by the sudden onset of orca calls, broadband synthetic sounds, and 500 Hz pure tones^21^.

In this study, we tested the effects of continuous sound playback on eight shark species that were attracted to an area using bait (potential food stimulus). We tested two sound stimuli in the wild on free-swimming sharks: (1) recordings of orca calls recorded in South Australia and (2) an artificial sound with 95% of its energy below 1 kHz. Although our sounds consisted of disjunct components (mixed frequencies and intensities), they were played continuously and did not contain periods of silence, i.e. there was no sudden onset of noise after the initial playback began. Therefore, we predicted an ‘orienting’ response (either towards or away) rather than a startle or a defence reaction, which may be expected following sudden presentation of a novel sensory stimulus^59^.

**Figure 1:**
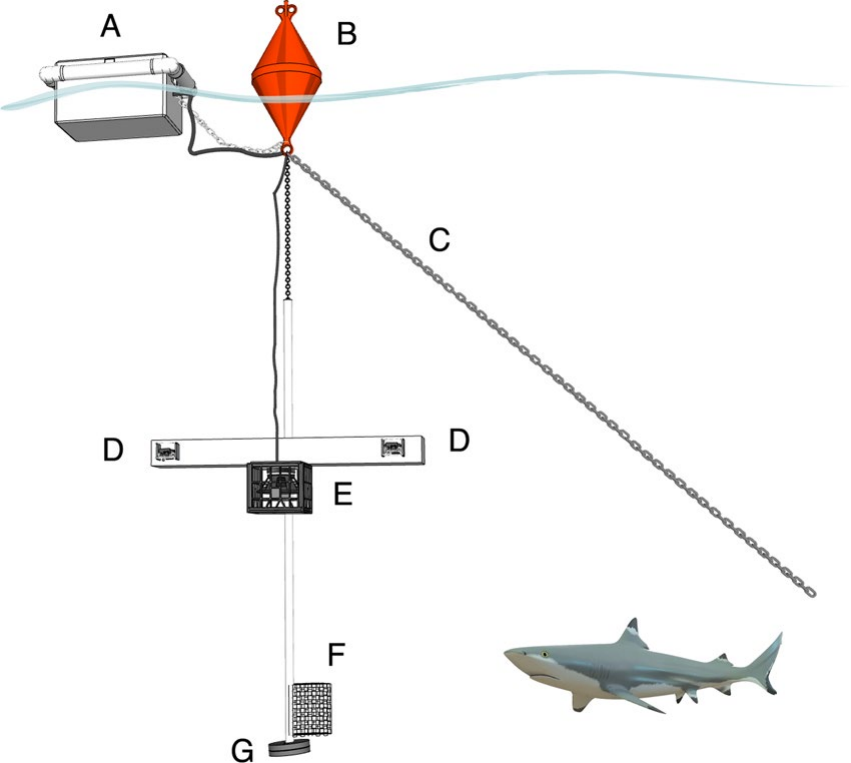
Schematic diagram of the stereo video camera system used for remotely monitoring shark behaviour and playback of sound. **A**: waterproof floating container for battery, power amplifier and MP3 player; **B**: surface buoy; **C**: anchoring chain to seabed; **D**: GoPro Hero3 video cameras; **E**: underwater speaker in protective plastic cage; **F**: bait bag, **G**: weights. Not to scale. Shark 3D model designed by KangarooOz 3D and used with permission from CGTrader.

Using an underwater camera rig (Fig. 1), we recorded the behaviour of white sharks (*C. carcharias*) and seven species of benthopelagic reef and coastal sharks: sicklefin lemon sharks (*Negaprion acutidens*), bronze whalers (*Carcharhinus brachyurus*), grey reef sharks (*Carcharhinus amblyrhynchos*), dusky sharks (*Carcharhinus obscurus*), sandbar sharks (*Carcharhinus plumbeus*), scalloped hammerhead sharks (*Sphyrna lewini*), and zebra sharks (*Stegostoma fasciatum*). Specifically, we examined the results of four scenarios: (i) whether either acoustic stimulus (presented separately) had an effect on the behaviour of the sharks encountered, and if the sharks were less likely to approach a baited camera while the sounds were playing; (ii) if the sounds had an effect on all species and/or if there were interspecific differences in the behavioural responses to each sound, (iii) individual behaviours of white sharks (*C. carcharias*) to each presented stimulus to assess any intraspecific variation; and finally, (iv) the incidence of tolerance to the introduced sounds as either an aversive or attractive stimulus. In each case, the control treatment comprised a non-functional speaker.

## Results

### Reef and coastal shark species (Order: Carcharhiniformes), Exmouth, Australia

Seven shark species were observed during behavioural trials in Western Australia: sicklefin lemon (*N. acutidens*), bronze whaler (*C. brachyurus*), grey reef (*C. amblyrhynchos*), dusky (*C. obscurus*), sandbar (*C. plumbeus*), scalloped hammerhead (*S. lewini*), and zebra (*S. fasciatum*) shark. A total of 533 interactions were recorded, of which nearly 90% were categorised as ‘passes’, and the remainder were classified as ‘touch rig’, ‘bump rig’, ‘bump bait’, ‘taste bait’ and ‘bite bait’ (Table 1).

**Table 1:** Summary of all data recorded from the reef and coastal sharks in Exmouth, Australia. Numbers in brackets are standard errors. N, number.

Parameters	All	Control	Orca Sound	Artificial Sound
**Reef and coastal sharks (7 species)**				
N of drops	67	32	16	18
N of interactions (total)	533	434	48	51
N of passes	478	384	46	48
N of touches	12	12	0	0
N of bumps rig	9	7	0	2
N of bumps bait	7	6	1	0
N of taste bait	18	17	1	0
N of bites	9	8	0	1
Total time on screen (s)	498	324	132	42
Mean time on screen (s)	15.4 (0.6)	15.7 (0.6)	11.5 (0.5)	17.4 (1.7)
Mean time of arrival (s)	1422 (168)	1578 (192)	864 (360)	2526 (396)
Mean total score	15.5 (3.0)	20.5 (4.5)	8.1 (3.1)	6.0 (1.8)

**Figure 2:**
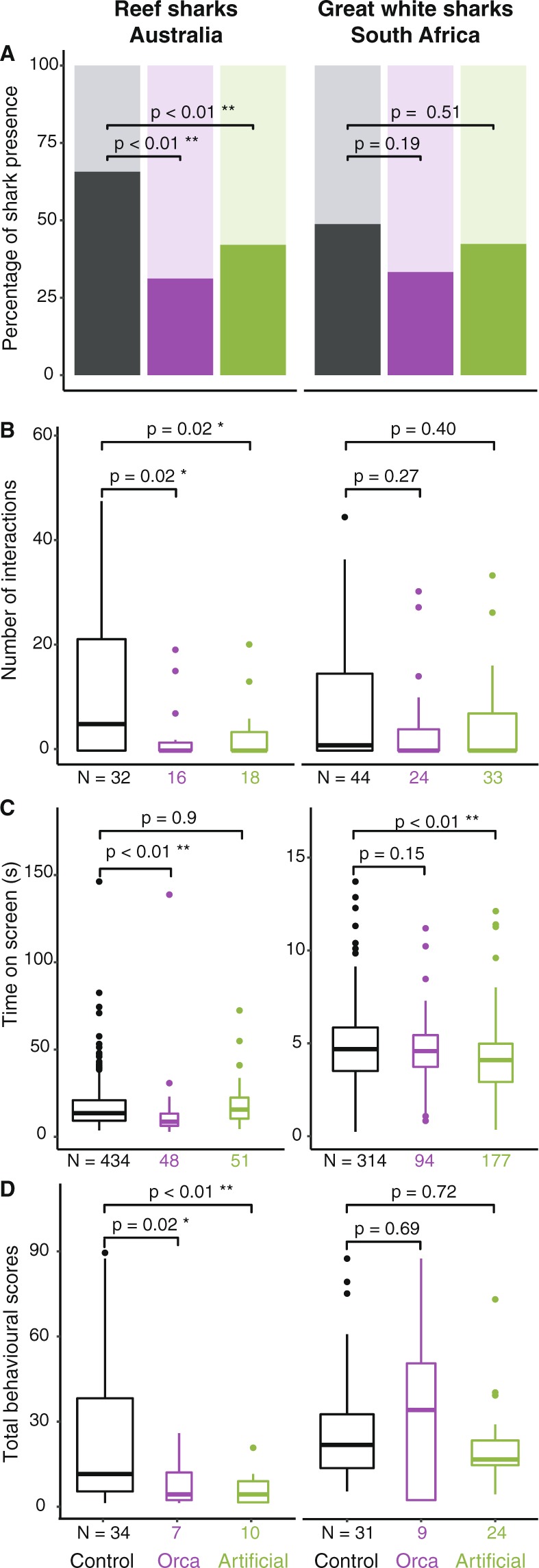
Plots representing the data for reef and coastal sharks (left panels) and for the white shark, *Carcharodon carcharias*, (right panels) conditional on treatments: Control (black), Orca Sound (purple) and Artificial Sound (green). (**A**) Proportion of presence (dark) and absence (light) of sharks observed for each drop. (**B**) Number of interactions. (**C**) Total time of shark interaction. (**D**) Total behavioural score (inquisitiveness). Significance is indicated (see Tables S2 and S3 for details) and boxplot width is adjusted for sample sizes (N).

When presented with either acoustic stimulus (orca or artificial sound), sharks approached the bait canister less frequently (Fig. 2A, Table S2, binomial GLMM, df = 1, orca: p < 0.01, artificial sound: p < 0.01). Similarly, the presentation of either sound treatment resulted in fewer interactions (orca: 48 interactions, artificial: 51 interactions) compared to the control (434 interactions) (Fig. 2B, Table S2, negative binomial GLMM, df = 1, orca: p = 0.02, artificial: p = 0.02). Sharks spent significantly less time in close proximity to the source of the acoustic stimulus when the orca sound was active (mean ± SD, time on screen 11.5 ± 0.5 s) compared to control trials (15.7 ± 0.6 s), although the artificial sound did not affect the time on screen (Fig. 2C, Table S2, gaussian GLMM, df = 3, orca vs control: p < 0.01, artificial vs control: p = 0.9).

Each species responded differently to the sounds; the amount of time that sharks spent in close proximity of the rig was affected significantly by the factor ‘species’ (Likelihood ratio test, χ2 = 113.53, df = 6, p < 0.01). *N. acutidens* and *C. plumbeus* were observed on screen more frequently than other species in all treatments.

It took on average 42.1 ± 6.6 min (mean ± SD) for the first shark of any species to appear on screen when the artificial sound was playing, which was significantly higher than the 26.3 ± 3.2 min observed when there was no sound playing (control treatment) (Table S2, gaussian GLMM, df = 3, p = 0.01). Again, there was a strong effect of the factor ‘species’ on the time of arrival (likelihood ratio test, χ^2^ = 17.455, df = 6, p < 0.01): *N. acutidens* and *C. brachyurus* were generally the first species to be observed. There was, however, no difference in the arrival time of the first shark when comparing the orca and control trials (Table S2, gaussian GLMM, df = 3, p = 0.18).

The different behaviours (‘pass’, ‘touch rig’, ‘bump rig’, ‘bump bait’, ‘taste bait’, ‘bite bait’) were translated into scores and summed, where higher scores corresponded to a greater level of physical interaction with the rig (see methods). The scores appeared significantly lower for both acoustic treatments (mean of sums across all species ± SD, 8.1 ± 3.1 for orca and 6.0 ± 1.8 for artificial sound) than for the control, which averaged 20.5 ± 4.5 (Table S2, Fig. 2D). Here too, ‘species’ was found to be a significant factor when placed as a fixed factor (likelihood ratio test, χ^2^ = 12.572, df = 6, p < 0.05). Again, *C. obscurus* was the highest scoring species (mean score ± SD, 1.74 ± 1.58, although they only interacted 82 times), closely followed by *N. acutidens* (mean score 1.71 ± 1.47 for a total of 432 interactions).

### White sharks, Mossel Bay, South Africa

Only *C. carcharias* were encountered in South Africa (Table 2). The two sound treatments did not affect the likelihood of observing a shark on camera (Fig. 2A, Table S3, binomial GLMM, df = 1, orca: p = 0.19, artificial: p = 0.51). Overall, we recorded 593 interactions, with ‘passes’ (behavioural score = 1) representing 45% of these (Table 2). The number of interactions was not influenced by either of the sound treatments compared to the control (Fig. 2B, Table S3, negative binomial GLMM, df = 1, orca: p = 0.27, artificial: p = 0.72).

**Table 2:** Summary of all data recorded from white sharks in Mossel Bay, South Africa. Numbers in brackets are standard errors. N, number.

Parameters	All	Control	Orca Sound	Artificial Sound
**White sharks (** ***Carcharodon carcharias*** **)**				
N of drops	101	44	24	33
N of interactions (total)	593	318	96	179
N of passes	271	146	39	86
N of touches	32	24	2	6
N of bumps rig	11	7	2	2
N of bumps bait	187	98	34	55
N of taste bait	74	38	13	23
N of bites	18	5	6	7
Total time on screen (s)	2718	1494	498	726
Mean time on screen (s)	4.3 (0.1)	4.5 (0.1)	4.5 (0.2)	3.9 (0.1)
Mean time of arrival (s)	2874 (282)	2802 (426)	4014 (426)	2478 (516)
Mean total score	25.3 (2.6)	27.3 (3.8)	32.1 (9.7)	20.1 (2.8)

However, *C. carcharias* spent significantly less time in proximity of the rig when the artificial sound was playing, while the orca sound had no effect (Fig. 2C, Table S3, gaussian GLMM, df = 3, orca: p < 0.01, artificial: p = 0.15). The time spent on screen was also significantly affected by the identity factor ‘ID’ (Likelihood ratio test, χ2 = 142.56, df = 38, p < 0.01). Overall, we identified 38 individual white sharks. The time of arrival of the first individual for each deployment was not significantly different between treatments (Table S3, gaussian GLMM, df = 3, orca: p = 0.5, artificial: p = 0.08). However, again, there was a strong effect of the IDs (likelihood ratio test, χ2 = 147.99, df = 38, p < 0.01). The behavioural scores were similar among treatments (Fig. 2D, Table S3, negative binomial GLMM, df = 1, orca: p = 0.69, artificial: p = 0.72). Here too, the IDs were found to be an important factor when considered as a fixed factor and some individuals scored significantly higher than others (Likelihood ratio test, χ2 = 123.65, df = 38, p < 0.01).

We tested whether the total time on screen changed significantly with repeated encounters with the equipment and found that ‘experience’ was significant as a fixed factor interacting with ‘time on screen’ (likelihood ratio test, χ2 = 13.428, df = 3, p < 0.01). Experienced sharks spent comparatively less time in the area when the artificial sound was playing with respect to the control (Table S3, gaussian GLMM, df = 3, control vs artificial: p = 0.05, control vs orca: p = 0.79). There was, however, no effect on the behavioural scores when the factor ‘experience’ was considered as a fixed factor (likelihood ratio test, χ2 = 37.5, df = 3, p = 0.54).

## Discussion

We investigated whether two sound stimuli altered the behaviour of sharks in the wild, using a baited downward facing midwater stereo-video system rigged with underwater speakers. An artificial sound and a recording of orca (*Orcinus orca*) calls were played back to seven different species of reef and coastal sharks around Exmouth, Western Australia, and to white sharks (*Carcharodon carcharias*) in Mossel Bay, South Africa. As a group, the reef and coastal sharks were found to behave differently in response to both sound treatments when compared to the control, with fewer sharks approaching the rig/bait and fewer interactions overall when the sounds were playing. There was also a decrease in behavioural scores (‘inquisitiveness’), as the sharks exhibited lower score behaviours (e.g. ‘pass’ rather than ‘bite’) when the sounds were playing. In addition, the orca sound decreased the overall time that the reef and coastal sharks spent on screen, and the artificial sound delayed the time of appearance. Likewise, *C. carcharias* took longer to arrive and spent less time on screen when the artificial sound was played back. However, the orca calls did not elicit a change in behaviour in *C. carcharias*. These results suggest species-specific differences in sensitivity and/or reactivity to certain types of auditory stimuli under the conditions tested.

Our results support earlier findings that certain underwater sounds can alter the behaviour of some sharks and potentially deter them from entering an area and/or interacting with a potential food source^20^. The natural soundscape of a shark comprises ambient sea noise that consists of both abiotic (wind, waves, etc.) and biotic (sounds made by marine organisms: mammals, fish, invertebrates) components^60–63^. The sounds perceptible to sharks (below 1.5 kHz) would mostly include continuous and/or rhythmic sounds, such as waves and bubbles, hydrodynamic flow of fish schools and the lower frequency components of some animal calls, like fish calls. An arrhythmic and chaotic sound (such as the artificial sound used in this study), with quick variations of intensities and frequencies, would represent an atypical and unfamiliar acoustic signal. This unnatural cue may trigger either investigative or aversive behaviour in some species of sharks.

**Figure 3:**
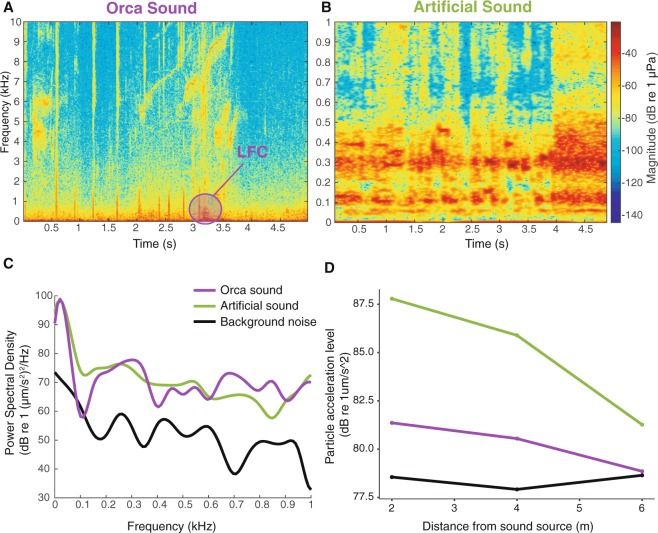
(**A**,**B**) Spectrograms of subsections of the field recordings of the orca sound (**A**) and artificial sound (**B**). LFC: low frequency component. Note the different frequency scale used for (**A**,**B**). (**C**) Power spectral density (PSD) for z-axis particle-acceleration levels of subsections of field recordings of artificial sound, orca sound and ambient background noise at calibration location, at a depth of 1.5 m. (**D**) Particle acceleration level spread loss with distance from sound source at calibration location, at a depth of 1.5 m.

Given that the particle acceleration level of the orca audio stimulus was smaller than that of the artificial sound (Fig. 3D), the amplitude may not have been high enough to trigger a response in *C. carcharias*. Although we cannot eliminate the possibility that *C. carcharias* are insensitive to these calls, this lack of reaction may also be due to the specific signature of the tested orca calls. Orca are known to have pod-specific calling behaviour and repertoire^64^ and even within-pod-specific call types^48^. Discriminatory antipredator behavioural responses to orca calls have been observed in harbour seals (*Phoca vitulina*), which responded strongly to calls of mammal-eating orcas but not to the calls of a local fish-eating population^53^. The white sharks of Mossel Bay in South Africa may not be reactive to the particular orca playbacks used in this study, whose signatures were recorded from a pod in South Australia, although it is currently unknown whether white sharks are sensitive to regionally-specific orca calls. The white sharks encountered in South Africa were, on average, larger (approximately 4 m in length) than the reef and coastal sharks (approximately 2–2.5 m) in Western Australia, which may be an easier target for orca.

Reef and coastal sharks spent relatively less time in the vicinity of the presented sounds when compared with white sharks and were also less likely to directly interact with the rig. We also found interspecific differences in behavioural responses between the reef sharks tested; the ‘species’ factor was a strong predictor of time spent in the area, time of arrival and the total behavioural scores. For example, we observed *N. acutidens* biting down on the bait and not releasing for a few seconds, while other species, such as *C. plumbeus* and *S. lewini* failed to touch the bait. Overall, *C. carcharias* appeared to be the most inquisitive of all the species encountered, having the most interactions with the bait and obtaining the highest behavioural scores.

Different shark species occupy different ecological niches and present a large array of life history traits: body size, habitat, mobility, diet and mode of reproduction are examples of factors that are highly variable in sharks^65^. Additionally, sharks have been shown to possess a large repertoire of complex behaviours and social displays^66–68^. In this study, the response to the rig and bait, as well as the playback speaker and type of sound, may be partially shaped by the combination of those life history traits. Specifically, the reaction to sound may be related to the characteristics of the soundscape niche occupied by different species of sharks. The habitat and its local ambient acoustics, and each species’ mode of locomotion and diet are potential factors that could shape the soundscape niche occupied by different species. As an example, we would expect reef and coastal sharks to live in a soundscape defined by the ambient sounds of the reef, characterised by invertebrates and fish sounds^69,70^, while white sharks (*C. carcharias*) would experience a diverse arrays of environments, from coastal soundscapes influenced by the dynamics of moving bodies of water, to open water environments with weather-related noise to complex rocky reef soundscapes.

Our findings not only suggest that species respond differently to auditory stimuli, but also that behavioural reactions to acoustic stimuli vary between individuals. Individual white sharks showed significant differences in the time spent around the rig and the scores of behaviours exhibited, independently of their prior experience to the rig. Intraspecific differences could be influenced by a range of factors, including sex, size, life stages, group dynamics and social hierarchy.

We also measured the level of tolerance (i.e. the intensity of disturbance that an individual tolerates without responding in a defined way^71^) that individual white sharks (*C. carcharias*) showed towards a stimulus after a few encounters (‘experience’), by observing the change in the time they spent in the vicinity of the speaker or the change in behaviour towards the rig and bait (scores). Contrary to our expectations, we did not observe any tolerance to the sensory stimuli over time. In fact, we observed that experienced white sharks spent comparatively less time in the area around the rig with the artificial sound playing than with the control treatment. After an initial inspection, some individuals may have refrained from further investigation due to the lack of food reward, or to the potential that the stimulus could be aversive. This process could lead to sensitisation, where the animals learn that a repeated or ongoing stimulus has significant consequences^72^. Each of the tests were run for only one hour, and over the course of 12 days, which is not sufficient to be able to explore the potential longer-term aspects of habituation and/or sensitisation. Nevertheless, our artificial sound was designed to have a constantly changing frequency and amplitude content that would perhaps be expected to reduce a potential tolerance and habituation effect.

Ultimately, the large variability shown in our results (Fig. 2) agrees with other studies investigating the effects of sounds and noise on marine fauna, where interspecific differences, intrapopulation variation, context of exposure and prior experience may change the responses of the animals to the stimulus^73^. For example, cooperative breeding cichlid fish species *Neolamprologus pulcher* exhibit sex-dependent behavioural responses to the same playback of boat noise^74^. Similarly, in a study exploring the responses of *Orcinus orca* to ship noise, the behavioural responses differed with the time of the year and the age of the animals^75^. Such variability might also be expected in the responses of apex predators like sharks, given that received sound or noise is only one of the many factors amongst a multimodal array of sensory cues that they must assimilate as they explore their environment^76^.

From a conservation perspective, it is concerning that some sharks changed their behaviour in response to a relatively low sound level (received levels at a distance of 2 m from source: Z-axis acceleration = 0.0245 m/s^2^, SPLrms = 150 dB re 1 μPa). Most anthropogenic sources (not only high intensity sources such as seismic air guns, pile driving and sonar, but also background noise like shipping) have much higher sound levels^2^. For example, McCauley *et al*.^77^ explored the effect of an airgun on pink snapper (*Pagrus auratus*), with an airgun which had a source level at 1 m of 203.6 dB re 1 μPa (SPLrms). The fish exposure to such a stimulus caused significant damage to the hair cells of the inner ear. Here, we have shown that a relatively low intensity sound played with a small underwater speaker is able to significantly modify reef shark behaviour. The frequency sensitivity of sharks overlaps with the range of anthropogenic noise, most of the latter lying in the low frequency range (<2 kHz)^2^. Although this study did not allow us to ascertain which of the temporal or the spectral attributes of a sound were most efficient in deterring sharks, previous observations on wild sharks showed it may be abrupt changes in amplitude levels rather than the spectral attributes of a sound that may trigger the behavioural responses. While this agrees with the general understanding that the fish hearing system has adapted as a temporal analyser, where the temporal patterns (rather than spectral) are the physiologically and behaviourally important parts of a sound^78^. This is in contrast to marine mammals, for which the spectral attributes of anthropogenic noise and the context of the animal (behavioural state at the time of exposure and demographic factors) act as a predictor for a withdrawal response, rather than the amplitude alone^75,79^. Considering the lack of knowledge of hearing physiology and acoustic behaviour in sharks^80^, we propose that there is a critical need for more studies on the impact of anthropogenic noise in cartilaginous fishes.

Although acoustic deterrents have proven successful in reducing bycatch of some marine organisms, such as cetaceans^81^, pinnipeds^6,82^ and bony fishes^12,13,83^, this study shows that further work is required to assess their efficacy with sharks. Species-specific and individual differences documented in behaviour, ecology, and the peripheral and central nervous systems^66,84–86^ suggest that one acoustic stimulus will be unlikely to deter all species of sharks equally. In fact, targeting more than one sensory modality may prove to be a better strategy^87^. Combinations of sounds, lights and bubbles, for example, which target the auditory, visual and lateral line systems of fishes, respectively, have proven successful in various applications^37,88–91^. There is also a substantial technical challenge in developing underwater acoustic repellents. The transducer must be large enough to produce low frequency components and provide enough energy and particle motion to spread several metres in order to affect the sharks’ auditory system, which, at present, is financially and technically challenging. With these constraints in mind, we are still far from the development of an individual acoustic repellent device that the public would be able to use in their daily aquatic activities or a beach-based mitigation device that can be attached to nets or longlines. However, confined areas like beaches could potentially be enclosed by repellent sounds and thus reduce the incidence of shark-human interactions and/or bycatch. In this context, the effect of such a sound on other marine organisms present in the area should be carefully considered. Nevertheless, in conjunction with underwater acoustic technological advancement, there may be a possibility of developing static, long-term management strategies for shark mitigation, especially if combined with another stimulus as a multimodal system.

## Methods

### Ethics statement

This study was carried out under the approval of The University of Western Australia Animal Ethics Committee (Application RA/3/100/1193) and in strict accordance with the guidelines of the Australian Code of Practice for the Care and Use of Animals for Scientific Purposes (8th Edition, 2013). The work was also approved by the Western Australia Department of Parks and Wildlife (Permits SF009446 & CE003980, 2014; SF010295 & CE004834, 2015) and the South African Department of Environmental Affairs: Biodiversity and Coastal Research, Oceans and Coasts Branch (Permit RES2014/91).

### Experimental rig

To record the behaviour of sharks in the wild, we used a downward facing midwater stereo-video camera system attached to a rig (Fig. 1) previously described by Kempster *et al*.^92^. Rigs were deployed from an anchored boat and suspended in mid-water with a pair of buoys at the surface. Once deployed, the boat left the area and the cameras recorded continuously for a maximum of 90 minutes. The sound device was composed of an underwater speaker (Diluvio from Clark Synthesis) positioned between the cameras pointed towards the bait (Fig. 1). The speaker was powered by a 12 V battery, through an automotive audio amplifier (PBR300X4; Rockford Fosgate), linked to an MP3 player (PhilipsGoGear). The MP3 player, amplifier and battery were enclosed within a waterproof container floating at the surface of the water, attached to the surface buoys, and the audio signal delivered to the submerged speaker via insulated electrical cables.

### Experimental design and sound treatments

The experiment involved the presentation of two different treatment sounds and one control treatment, comprising a similar but non-functional speaker and floating container. The first sound treatment was an artificially produced sound (referred to as the ‘artificial sound’), composed with Adobe Audition CS5.5, the digital audio workstation software Reaper v.42 (Cockos Inc.), and the virtual instrument Granite (New Sonic Arts Inc.) as an audio unit instrument plug-in. The sound consisted of mixed tones of different intensities and frequencies, from 20 Hz to 10 kHz, but with 95% of the energy at frequencies lower than 1 kHz (Fig. 3B) to fall within the presumed shark hearing frequency range. The artificial sound contained most of its power in the lower frequencies (overlapping with the peak sensitivity of the auditory system in sharks), chaotic rhythms (i.e. abrupt changes, no temporal domain pattern) and sudden increases and decreases in intensity (Fig. 3B). With the aid of the granular texture generator (Granite, New Sonic Arts Inc.), we used a technique known as granular synthesis^93–95^ to build the sound, which allowed us to randomly alter the temporal domain without changing the desired frequency (pitch) to restrict to the low range^96^. A 30 second extract of the artificial sound is provided as Supplemental Information (S5).

The second sound treatment consisted of a combination of orca calls (*Orcinus orca*) (referred to as ‘orca’) (Fig. 3A). Orca populations usually specialise in one or several prey species of mammals, penguins or fishes^97,98^ and their acoustic behaviours are known to vary with the type of prey hunted^99^. More specifically, groups of killer whales communicate extensively while hunting bony fishes^99,100^. The calls used as playback in this study were recorded by David and Jennene Riggs (Riggs Australia, www.riggsaustralia.com) in South Australia in February 2014 within a mile of a pod of approximately 20–30 individuals. Although the limited information on the distribution, movements, and population status of Australian orca do not allow us to unequivocally class the observed pod as a shark-eating population, we have selected calls that were recorded prior to a mass predation on ocean sunfish (*Mola ramsayi*). The pattern of the recorded pulsed calls would thus represent calls typically used by the pod when predating on large fishes. Although orca calls can peak to about 25 kHz, we preferentially selected low frequency components (LFC, <1000 Hz) in the recordings (Fig. 3A). For both sounds, we built a recording of 15 minutes duration (of unique sound waves), which we repeated four times to make two final sound files of an hour each.

### Calibration of sound device

The sound device was calibrated in a calm location in a river to avoid boat noise (Swan River, Western Australia, salinity 33 ppt, temperature 21 °C, average depth 3.5 m). The speaker was deployed at a depth of 3 m and both sounds were played back and recorded at a depth of 1.6 m with two HTI 90 U hydrophones (High Tech, Inc.), where responses were considered linear from 2 Hz–20 kHz. The recordings were made with a NI USB 6353 Data Acquisition device (National Instruments), and the system gain was estimated with a white noise calibrator. The sounds were measured at three distances (2 m, 4 m and 6 m) from the speaker, to estimate propagation loss. All parameters were calculated with a custom-made code in MATLAB (2017a, The MathWorks, Inc.) (Table S1). Particle accelerations were estimated from pressure gradient measurements using the Euler equation, as described by Mann^101^ (Table S1, Fig. 3C) with two hydrophones mounted in an array. The magnitude of the particle acceleration was calculated as the sum of the squared accelerations for each axis (Table S1). Figure 3D shows the loss of the particle acceleration magnitude, with the distance from the underwater speaker. This calibration could not be considered as an exact reference for all our data, as field conditions were not consistent each day and at each location, which would have impacted the acoustic behaviour and propagation loss of the sound device. However, we considered these sound parameters to represent a general reference for stimulus magnitude.

To determine the effect of any electronic signal emanating from the speaker, the electrical signature of the sound device was characterised as described and reported by Ryan *et al*.^37^. The artificial sound and the orca sound produced a peak-to-peak signal of 0.115 mV and 0.118 mV, respectively. Therefore, we considered that the electrical signature of the speaker would not significantly influence the behaviour of the sharks encountered, unless they came within 30 cm of the speaker, which never occurred in the sound (active) treatments.

### Field sites

The fieldwork was conducted in two distinct areas in order to target different shark species. The effects of sound stimuli on reef and coastal shark species were investigated in Exmouth, Western Australia, where three field sites were visited over a period of seven days in April 2014, and five days in April 2015: VLF Bay, Burrows Reef and North West of the Murion Islands (Fig. S1A). Overall, 70 deployments of stereo camera rigs (35 control, 16 orca, 19 artificial sound; 28 at Burrows Reef, 34 at VLF Bay, 8 at NW Murion) were carried out offshore from Exmouth, at depths ranging from 12–19 m (mean = 14.1 ± 1.75 m).

The effects of sound stimuli on white sharks were investigated in Mossel Bay, South Africa, where two field sites were visited over a period of 12 days in June 2014: Seal Island and Hartenbos river mouth (Fig. S1B). During deployment of the video-camera rigs, additional bait (chum) was introduced into the water to attract white sharks into these areas. Overall, 101 deployments were carried out in South Africa (44 control, 24 orca, 33 artificial sound; 58 at Hartenbos, 43 at Seal Island), at depths ranging from 10–18 m (mean = 15.0 ± 1.6 m).

### Video analysis

Only the 60 minutes of treatment time on the video footage was analysed. The white sharks in South Africa were each identified by individual markings, scars and dorsal fin profiles^102^. Since no such obvious markings could be identified in the reef and coastal sharks in Western Australia, individuals could not be discriminated. The data acquired from the video analysis consisted of species, individual shark IDs (for white sharks), time of arrival, total time the shark was present in the field of view of the cameras (on screen), number of interactions, and notable behaviours. A shark swimming by, present in the field of view, was accounted as an ‘interaction’, even though it did not physically ‘interact’ with the rig/bait. Observed behaviours for each interaction were classified into one of six categories: (1) ‘pass’ (shark in the field of view but did not make contact with the rig), (2) ‘touch rig’ (shark touched any part of the rig), (3) ‘bump rig’ (shark touched the rig elsewhere than the bait bag or canister, with snout), (4) ‘bump bait’ (shark touched the bait bag or canister with snout), (5) ‘tastes bait’ (shark touched the bait canister with an open mouth), and (6) ‘bite bait’ (shark was observed to make full contact with the bait banister in the form of a bite). We scored these behaviours in a progression of interactivity from 1–6, the lowest interaction being a ‘pass’ (scoring a 1) and the highest a ‘bite bait’ (scoring a 6).

### Data analysis

The effect of the sound treatments was determined using mixed model analyses performed in R^103^ with the packages ‘lme4’^104^ and ‘glmmADMB’^105^. The treatment was set as a fixed factor and the date, field site, time of day (morning, midday, afternoon), trials (matching treatment and control) were random factors. For the Mossel Bay dataset, shark identity (ID) and the number of previous interactions with the rig (‘experience’) were added as random factors. To determine if the presence or absence of sharks on the footage was defined by treatments, we performed a binomial generalised linear mixed model (GLMM). To investigate if there were differences in the number of interactions per treatment, we started with a Poisson general linear model (GLM) and obtained non-linear residual patterns and overdispersion. We then fitted a Negative Binomial GLM and verified levels of independence. The same model was used to test the scores. In some cases, transformation of the time data was required (see Tables S2 and S3 for details) to achieve linearity of the residuals. A stepwise procedure was used to consider all possible combinations of predictors and lowest Akaike Information Criteria (AIC) was used to select the final models. To determine the difference between treatments, a multiple comparison for parametric models was performed using the R package ‘multcomp’^106^. All statistical plots presented in this study were designed with R package ‘ggplot2’^107^.

## Supplementary information

Supplementary Dataset 1

## Data Availability

The datasets generated and analysed during the current study are available from the corresponding author on reasonable request.
