# Supplementary material for: The effect of underwater sounds on shark behaviour
Source: Sci Rep. 2019 May 6;9:6924. doi: 10.1038/s41598-019-43078-w (PMC6502882; doi:10.1038/s41598-019-43078-w)
Supplement: Supplementary file 1 — Supplementary Dataset 1 [file 41598_2019_43078_MOESM1_ESM.docx]

**The effect of underwater sounds on shark behaviour**

**Lucille Chapuis^1,2,*^, Shaun P. Collin^1,3^ , Kara E. Yopak^4^, Robert D. McCauley^5^, Ryan M. Kempster^1^, Laura A. Ryan^6^, Carl Schmidt^1^, Caroline C. Kerr^1^, Enrico Gennari ^7,8,9^, Channing A. Egeberg^1^, Nathan S. Hart^6^**

- Correspondence: l.chapuis@exeter.ac.uk

1. Oceans Graduate School and the UWA Oceans Institute, The University of Western Australia, Perth, WA 6009, Australia
2. Biosciences, College of Life and Environmental Sciences, University of Exeter, Exeter, EX4 4QD, UK
3. School of Life Sciences, La Trobe University, Bundoora, VIC 3086, Australia
4. School of Biology and Marine Biology and the Centre for Marine Science, University of North Carolina Wilmington, Wilmington, NC 28403 USA
5. Centre for Marine Science and Technology, Curtin University, Perth, WA 6102 Australia
6. Department of Biological Sciences, Macquarie University, North Ryde, NSW 2109, Australia
7. Oceans Research, Mossel Bay 6500, South Africa
8. South African Institute for Aquatic Biodiversity, Grahamstown, 6139, South Africa
9. Department of Ichthyology and Fisheries Science, Rhodes University, Grahamstown, 6139, South Africa

**Supplemental Information**

**Figure S1.** Map of the deployment areas in Exmouth, Western Australia (A) and Mossel Bay, South Africa (B). AU: Australia, ZA: South Africa; 1: Burrow Reef; 2: VLF Bay; 3: NW Murion; 4: Seal Island; 5: Hartenbos River mouth. Map data: © 2016 Google.

**
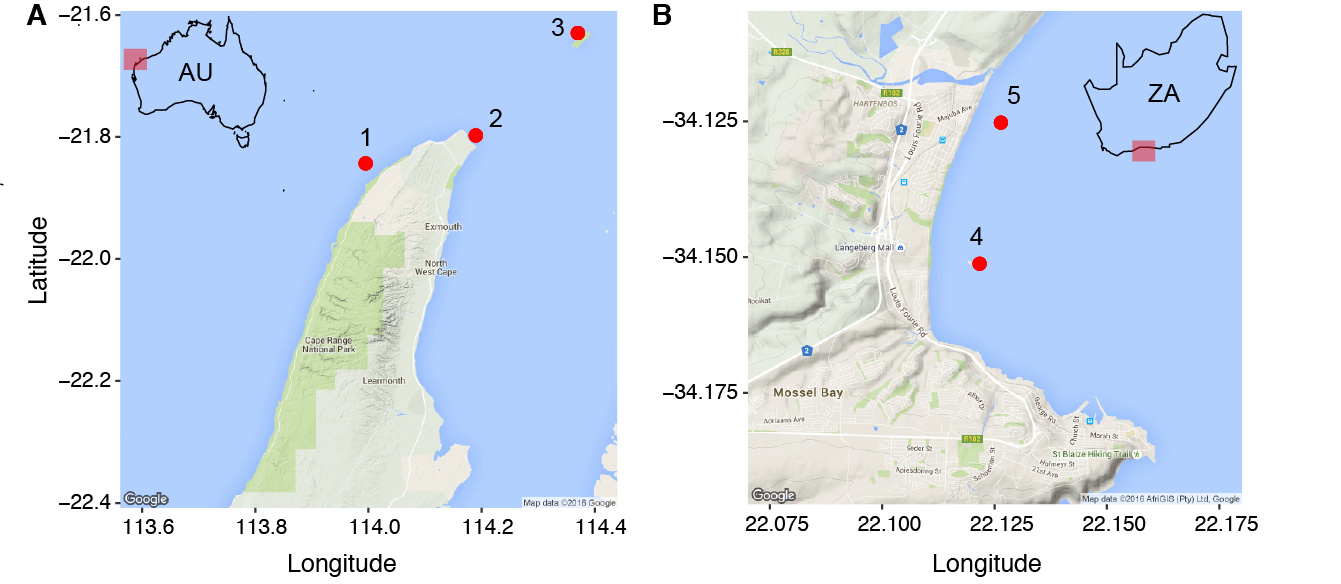
**

**Table S1.** Sound parameters for Artificial Sound and Orca at a distance of 2 m, 4 m, and 6 m from the speaker and the background noise recorded at the calibration location, at a depth of 1.5 m. Sampling rate: 10 kHz.

| **Sound parameters** | **Background noise** | **Artificial Sound, 2 m** | **Artificial Sound, 4 m** | **Artificial Sound, 6 m** | **Orca, 2 m** | **Orca, 4 m** | **Orca, 6 m** |
| --- | --- | --- | --- | --- | --- | --- | --- |
| RMS (Pa) | 9.38 | 38.07 | 30.50 | 7.62 | 36.61 | 29.86 | 7.92 |
| SPLpeak + (dB re 1µPa) | 146.11 | 158.95 | 155.75 | 148.83 | 158.95 | 154.71 | 155.11 |
| SPLpeak – (dB re 1µPa) | 131.51 | 151.63 | 125.37 | 145.27 | 138.46 | 139.56 | 147.21 |
| SPLpp (dB re 1µPa) | 277.62 | 310.59 | 281.12 | 294.11 | 297.42 | 294.28 | 302.32 |
| SPLrms (dB re 1µPa) | 139.44 | 150.81 | 149.69 | 137.63 | 151.27 | 149.50 | 137.98 |
| SELcum (dB re 1µPa^2^) | 155.74 | 164.62 | 162.70 | 150.65 | 164.28 | 162.51 | 150.98 |
| SNR (dB SNR) | -- | 12.15 | 10.24 | -1.81 | 11.83 | 10.06 | -1.46 |
| **Particle acceleration:** |  |  |  |  |  |  |  |
| X-axis accel. (m/s^2^) | 0.0108 | 0.0163 | 0.0118 | 0.0103 | 0.0117 | 0.0106 | 0.0088 |
| Y-axis accel. (m/s^2^) | 0.0075 | 0.0183 | 0.0123 | 0.0096 | 0.0147 | 0.0120 | 0.0115 |
| Z-axis accel. (m/s^2^) | 0.0086 | 0.0245 | 0.0197 | 0.0116 | 0.0136 | 0.0130 | 0.0128 |
| Magnitude of acceleration (m/s^2^) | 2.45E-04 | 1.20E-03 | 6.79E-04 | 3.32E-04 | 5.38E-04 | 4.28E-04 | 3.73E-04 |
| RMS, Root mean square; SPLpeak+, Sound pressure level of the highest positive peak; SPLpeak-, Sound pressure level of the highest negative peak; SPLpp, Sound pressure level of the peak to peak; SPLrms, sound pressure level of the root mean square value; SELcum, Sound exposure level cumulative; SNR, signal-to-noise ratio. | | | | | | | |

**Table S2.** Results of mixed models for experiments on reef and coastal sharks in Exmouth, Australia. Treatments considered as fixed effects: Control, Orca Sound, Artificial Sound. The first table shows the results for the binomial models and the lower table shows the results for the Gaussian models with multiple comparisons. Stars indicate significance: *: p ≤ 0.05, **: p ≤ 0.01. The sign † indicates a transformation of data. Model selection for each response is shown on the right side of the table, with delta Akaike Information Criterion (ΔAIC) and AIC weights.

| **Model** | **Response** | **Random effects** | **Factor** | **Estimate ± SE** | **DF** | **Wald’s Z** | **P-value** | **Model selection** | **ΔAIC** | **AIC weight** |
| --- | --- | --- | --- | --- | --- | --- | --- | --- | --- | --- |
| Binomial GLMM | Presence/ Absence of sharks | Area, time of the day, date | Intercept | 2.92 ± 1.48 | 1 | 1.98 | 0.04 * | response + area + time of day + date | 0.0 | 1 |
|  |  |  | Orca | -2.39 ± 0.8 |  | -2.97 | < 0.01 ** | area + time of day + date | 18.0 | < 0.001 |
|  |  |  | Artificial Sound | -3.17 ± 1.02 |  | -3.1 | < 0.01 ** |  |  |  |
| Negative binomial GLMM | Total number of interactions | Area, time of the day, date | Intercept | 1.67 ± 0.78 | 1 | 2.16 | 0.03 * | response + area + time of day + date | 0.0 | 0.97 |
|  |  |  | Orca | -1.39 ± 0.59 |  | -2.35 | 0.02 * | area + time of day + date | 6.7 | 0.03 |
|  |  |  | Artificial Sound | -1.36 ± 0.58 |  | -2.34 | 0.02 * |  |  |  |
| Negative binomial GLMM | Behavioural score | Area, date, species | Intercept | 3.14 ± 0.18 | 1 | 17.3 | < 0.01 ** | response + area + date + species | 2.0 | 0.27 |
|  |  |  | Orca | -1.04 ± 0.45 |  | -2.3 | 0.02 * | response + area + date | 6.8 | 0.02 |
|  |  |  | Artificial Sound | -1.35 ± 0.39 |  | -3.4 | < 0.01 ** | area + date + species | 0.0 | 0.71 |

| **Model** | **Response** | **Random effects** | **Factor** | **Estimate ± SE** | **Comparisons** | **DF** | **P-value** | **Model selection** | **ΔAIC** | **AIC weight** |
| --- | --- | --- | --- | --- | --- | --- | --- | --- | --- | --- |
| Gaussian GLMM | Time on screen (log) † | Area, date, time of the day, species | Intercept | -0.73 ± 0.10 | Orca vs Control | 3 | < 0.01 ** | response + area + date + time of the day + species | 0.0 | 0.986 |
|  |  |  | Orca | -0.23 ± 0.05 | Artificial vs Control |  | 0.9 | area + date + time of the day + species | 8.5 | 0.01 |
|  |  |  | Artificial Sound | 0.002 ± 0.04 | Artificial vs Orca |  | < 0.01 ** | response + area + date + time of the day | 40.2 | 0.0 |
| Gaussian GLMM | Time of arrival | Area, date, time of the day, species | Intercept | 36.72 ±5.37 | Orca vs Control | 3 | 0.18 | response + area + date + time of the day + species | 1.0 | 0.38 |
|  |  |  | Orca | 8.41 ± 6.09 | Artificial vs Control |  | 0.01 * | area + date + time of the day + species | 13.9 | <0.001 |
|  |  |  | Artificial Sound | 14.92 ± 6.16 | Artificial vs Orca |  | 0.41 | response + area + date + time of the day | 0.0 | 0.62 |

**Table S3.** Results of mixed models for experiments on *Carcharodon carcharias* in Mossel Bay, South Africa. Treatments considered as fixed effects: Control, Orca Sound, Artificial Sound. The first table shows the results for the binomial models and the lower table shows the results for the Gaussian models with multiple comparisons. Stars indicate significance: *: p ≤ 0.05, **: p ≤ 0.01. The sign † indicates a transformation of data. Exp, experience. Model selection for each response is shown on the right side of the table, with delta Akaike Information Criterion (ΔAIC) and AIC weights.

| **Model** | **Response** | **Random effects** | **Factor** | **Estimate ± SE** | **DF** | **Wald’s Z** | **P-value** | **Model selection** | **ΔAIC** | **AIC weight** |
| --- | --- | --- | --- | --- | --- | --- | --- | --- | --- | --- |
| Binomial GLMM | Presence/ Absence of sharks | Area, time of the day, date | Intercept | 2.7e-16 ± 0.30 | 1 | 0 | 1 | response + area + time of day + date | 2.2 | 0.25 |
|  |  |  | Orca | -0.69 ± 0.53 |  | -1.31 | 0.19 | area + time of day + date | 0.0 | 0.75 |
|  |  |  | Artificial Sound | -0.31 ± 0.46 |  | -0.66 | 0.51 |  |  |  |
| Negative binomial GLMM | Total number of interactions | Area, time of the day, date | Intercept | 2.09 ± 0.35 | 1 | 5.8 | < 0.01 ** | response + area + time of day + date | 2.6 | 0.22 |
|  |  |  | Orca | -0.67 ± 0.60 |  | -1.11 | 0.27 | area + time of day + date | 0.0 | 0.78 |
|  |  |  | Artificial Sound | -0.46 ± 0.55 |  | -0.85 | 0.40 |  |  |  |
| Negative binomial GLMM | Behavioural score | Area, time of the day, date, ID | Intercept | 0.96 ± 0.05 | 1 | 16.49 | < 0.01 ** | response + area + time of day + date + ID | 1.8 | 0.18 |
|  |  |  | Orca | 0.04 ± 0.39 |  | 0.39 | 0.69 | area + time of day + date + ID | 0.0 | 0.43 |
|  |  |  | Artificial Sound | -0.29 ± 0.36 |  | -0.36 | 0.72 | response area + time of day + date | 0.2 | 0.39 |

| **Model** | **Response** | **Random effects** | **Factor** | **Estimate ± SE** | **Comparisons** | **DF** | **P-value** | **Model selection** | **ΔAIC** | **AIC weight** |
| --- | --- | --- | --- | --- | --- | --- | --- | --- | --- | --- |
| Gaussian GLMM | Time on screen | Area, date, ID, experience | Intercept | 0.07 ± 0.01 | Orca vs Control | 3 | 0.46 | response + area + date + ID + experience | 0.0 | 0.48 |
|  |  |  | Orca | -0.005 ± 0.007 | Artificial vs Control |  | < 0.01 ** | response + area + time of day + date | 11.2 | 0.13 |
|  |  |  | Artificial Sound | -0.02 ± 0.005 | Artificial vs Orca |  | 0.15 |  |  |  |
|  | Time on screen x Experience | Area, date, ID, experience | Intercept | 0.069 ± 0.007 | Exp:Ctrl vs Exp:Orca | 3 | 0.79 | response x experience + area + date + ID | 3.3 | 0.39 |
|  |  |  | Orca | 0.001 ± 0.008 | Exp:Ctrl vs Exp:Artificial |  | 0.05 * |  |  |  |
|  |  |  | Artificial Sound | -0.015 ± 0.006 | Exp:Orca vs Exp:Arificial |  | 0.84 |  |  |  |
|  |  |  | Exp | 0.007 ± 0.0002 |  |  |  |  |  |  |
|  |  |  | Orca: Exp | -0.0008 ± 0.0004 |  |  |  |  |  |  |
|  |  |  | Artificial: Exp | 0.0003 ± 0.0003 | **Wald’s Z** |  |  |  |  |  |
| Gaussian GLMM | Time of arrival † (√) | Area, date, time of the day, ID | Intercept | 1.42 ± 1.12 | 1.27 | 3 | 0.20 | response + area + date + time of the day + ID | 0.0 | 0.43 |
|  |  |  | Orca | -0.61 ± 0.91 | -0.67 |  | 0.50 |  |  |  |
|  |  |  | Artificial Sound | -2.03 ± 1.18 | 1.722 |  | 0.08 | area + date + time of the day + ID | 1.6 | 0.57 |
